# On the Connexion between Morbid Physical and Religious Phenomena

**Published:** 1856-01-01

**Authors:** J. F. Denham


### ART. III.—ON THE CONNEXION BETWEEN MORBID PHYSICAL AND RELIGIOUS PHENOMENA.

NO. 5 OF A SERIES.

BY THE REV. J. F. DENHAM, M.A., F.R.S., ETC.

THE present paper will contain an account of those co-existing morbid physical and religious phenomena which the writer considers to have come under his notice, belonging to the department of *Remorse.* His design renders it requisite,—(1) to ascertain what is to be understood by genuine remorse; (2) to describe the characteristics of spurious remorse; (3) to adduce reasons for assigning to them a merely morbid and physical origin; (4) to suggest means of prevention and cure.

(1) By genuine remorse, according to the definition of it given by Clarendon, Bishop Hall, &c., and derived from the etymology of the word, is meant, "the keen pain or anguish excited by a sense of guilt; compunction for a vicious, or sinful act committed." Dr. Thomas Brown describes it as "the dreadful moral regret arising from the certainty that we have rendered ourselves unworthy of the love of man and of the approbation of our God"—"the salutary influence of conscience, which, though it cannot restore innocence itself, may at least, by the images which it awakes, soften the mind to that repentance which is almost innocence under another form,"—"that terrible voice which it is impossible to fly, because it is with us wherever we may fly, and which we can still only in one manner; by acting so as to merit, not its silence only, but its applause."* St. Paul defines it as the accusing testimony of conscience, involving both the existence of the moral "law written in the hearts of all men," and the painful consciousness of having transgressed that law:†

* "Lectures on the Philosophy of the Human Mind." Edinburgh. 1830, pp. 428, 233, 633.

† Rom. ii. 14, 15.

as "godly sorrow, working repentance unto salvation not to be repented of:*—literally, *sorrow according to God,* "a sorrow arising from causes out of which God would have it arise, and which have the effects he wishes them to have."† According to these definitions, genuine remorse is not only painful, but is also intelligent, pious, and curative, or promotes moral improvement; "is operative, diligent, and instrumental to caution and strict walking—the mother of holy living."‡ We must therefore (2) consign to the department of mere spurious remorse all those feelings, &c., which though in the latitude, or through the imperfection of language, commonly passing under the name of Remorse, have not these qualities nor tend to these results; but have only a distorted or inefficient semblance of them, amounting only to a mere *rue* (rudo), and consisting only of indefinable "low spirits," dread, anxiety, vexation, hypochondriacal grief and woe; and attended with a general torpor or debility of the judgment. The distinction between genuine and spurious remorse is intimated in the New Testament by the restriction of the term *μετάνοια*, or *reform,* to the former, and *μεταμέλεια, a mere sorrow,* to the latter; "because every one who reforms, repents; but every one who repents, does not reform."§ The Romans also called the former *conscientia, pietas;* the latter *furiæ, morsus, emorsus.* Horace thus describes a good repentance.

"Scelerum si *bene* pœnitet,

Eradenda cupidinis

Pravi sunt elementa; et teneræ nimis

Mentes asperioribus

Formandæ studiis."||

The prophet Hosea describes the subjects of spurious remorse as "not crying unto God with their heart, when they howled on their beds: as returning, but not to the Most High,"¶ the distinction being here made, as, in regard of the mere semblance of virtues, elsewhere, "not unto me,"** having neither pious motives, nor producing moral reformation.

(3) The following reasons are offered for assigning to the phenomena of spurious remorse a merely morbid and physical origin:—

I. It may be observed, generally, that spurious, or unintelligent, irrational, and unimproving remorse in the abstract, or when arising from even innocent causes, is never unassociated with either positive indications of bodily disease, or with a morbid

* 2 Cor. 7, 10, 11. † Rosenmüller, Schol. in loc.

‡ Bishop Jer. Taylor. Sermon 3, on Godly Fear. Part. i.

§ Campbell, "Diss." 6. || Carmen, Od. xxiv. lib. 3.

¶ Ch. vii. 14, 16. ** Zech. vii. 5.

diathesis of the body, loose fibre, sensitive and imaginative temperament, or at least, with a diminished state of physical power. The mildest form of it is the penitential regret experienced along with fatigue, arising, especially, from over-excitement, or from an unusual degree of innocent enjoyment; as in the evening or morning after "a day's pleasure," or during the homeward journey from a tour, or an excursion. It increases with the increase of disease; declines with convalescence: and, except in hypochondriacal temperaments, wholly ceases with the restoration of health and strength. In such temperaments it is liable to be revived by whatever excites the nervous system. Its exacerbations follow a full meal, are greatest at night and least in the morning; are mitigated by cathartic medicines and other means of alleviating hypochondriacal disorders.

II. Paroxysms of spurious remorse peculiarly follow the grosser sins of the flesh; as when the transgressor awakes in the morning, "*cœna desurgat dubia*," and becomes conscious of the excesses and irregularities of the preceding evening, and has, what in popular language are called, "the horrors,"—

"quin corpus onustum
Hesternis vitiis animum quoque prægravat unà,"

is ashamed of himself; and vexed at the disability of mind, body, and estate, which he has incurred. His desponding or excited emotions are, however, all connected, either with the stomach, which Haller justly calls "the conscience of the body," or with the heart, or with the brain; and generally partake more or less of delirium tremens:—are alleviable, and, perhaps, for many successive occasions, by alcoholic stimulants, and by other means which indicate the merely physical origin and nature of such emotions. Spurious remorse, indeed, in any violent degree, rarely follows immediately upon any depravity wholly unconnected with debauchery.

III. It is, also, seldom permanent until the physical powers have become, by such means, permanently impaired. Valetudinary sensualists have often remarked to me, that they experienced only temporary and trifling visitations of what they called remorse, until their health had become seriously damaged or completely undermined.

IV. Spurious remorse, however intense, produces no lasting reformation of conduct, gathers no improvement from religious motives, or even from considerations of self-interest. Its tendency is rather to weaken the moral powers and the judgment, by occupying the mind's attention with the physical feelings. Thus, the sensualist who, under the actual endurance of his miserable "next morning" sensations, may make resolutions, vows, promises—utter profuse self-condemnation and impassioned prayers, may, nevertheless, too often, be found to incur, perhaps in the evening of the same day, or at no distant period, a renewal of all his sufferings and humiliations, and thus to exemplify the Proverb that "as a dog returneth to his vomit, so a fool returneth to (margin, iterateth,) his folly:"* and to illustrate St. Peter's comparison of him to "the sow that was washed and turneth to her wallowing in the mire."† The following case of such fruitless remorse affords a specimen of an occurrence too frequent in the observation of all ministers of religion. "A man having upon his bed of sickness received in his own conceit the sentence of death in himself; and being pressed to humiliation and broken-heartedness, for he had formerly been a stranger and an enemy to purity and the power of godliness, answered thus: 'My heart is broken;' and so broke out into an earnest confession of particular sins; he named uncleanness, stubbornness, hypocrisy, &c. He compared himself to the thief upon the cross. "And if God," saith he, "restore me to health again, the world shall see what an altered man I will be." When he was pressed to sincerity and true-heartedness in what he said, he protested that he repented with all his heart and soul, and mind, and bowels, &c.; and desired a minister that stood by to be a witness of these things between the world and him. And yet this man upon his recovery became the very same, if not worse than he was before."‡

V. Spurious remorse frequently evinces its morbid origin by its being excited by the most trifling, absurd, and even imaginary causes, and by its disproportion to the cause of it, and not unfrequently by its being attended with an insensibility to real and even heinous ill-conduct on the part of the subject of it. I have met with cases *approximating* to that of the shepherd in Italy, who in his confession in Lent expressed his concern, that in making cheese, some of the milk had spurted into his mouth, and desired absolution for it, but who, upon being questioned by the priest whether he was not a party in those robberies and murders which were committed in the neighbourhood on travellers, readily owned it, and added that this mode of enriching themselves was not looked upon as criminal among his neighbours.§ A female of declining age, feeble health, uncultivated mind,—the subject of vivid religious emotions, strictly temperate, but guilty of ingenious cruelty towards a step-daughter, suffered intense remorse, at times, till the day of her death, from having, in haste, torn a leaf out of her Bible for the purpose of lighting

* Prov. xxvi. 11. † 2 Pet. ii. 22.

‡ Bolton, "On Affliction of Conscience," sect. 2, Part i. ch. 8.

§ De la Roche, "Mem. of Literat. for 1712."

a candle; feared that she had thereby "committed the sin against the Holy Ghost," &c. I find the following case of perverted remorse in my memoranda. A man aged nearly seventy, of tall stature, thin habit of body, dark melancholic complexion, sensitive, humane, intelligent, temperate, but *living in cohabitation*—and having lived so for several years, was during many months of his last bedridden illness in a narrow, dark, and unwholesome apartment, evidently suffering from deep-seated remorse. Upon being urged to "open his grief," he said, "I have, indeed, something on my mind. In my younger days I was a soldier, and served in the West Indies. While we were there, the troops thought themselves hardly used. Some of them mutinied. One of them shot an officer, and was sentenced by the court-martial to be hanged. An offer was made of thirty shillings to any one who would act as executioner. I accepted the offer, and since I have been lying here, the thought of it comes into my mind day and night. I think I did wrong. The recollection of it distresses me more than anything I ever did beside." He repeated the name of the soldier very mournfully, "Poor Joseph!" It appeared to me that his mind had conceived a mal-association with his deed, partly, at least, from the similarity of the sum of money to that received by Judas Iscariot for the betrayal of his master. He was much inclined to superstition, and told stories of what he considered to be retributions of Divine Providence *in kind*, which he had witnessed, in which, however, the resemblances seemed to be indistinct. Nor could he derive lasting consolation from any moral reasoning offered to him respecting the particular cause of his own mental sufferings—could not be made sensible that he was living in sin.

VI. The irrational and disproportioned nature of mere spurious remorse is thus depicted by one of the most eminent Puritan divines. "In all other adversities a man is still a friend unto himself, favours himself, and reaches out his best considerations to bring in comfort to his heavy heart. But in this he is a scourge to himself; at war with himself; an enemy to himself. He doth greedily and industriously fetch in as much matter as he can possibly, both imaginary and true, to enlarge the rent and aggravate his horror. He gazes willingly in that false glass which Satan (?) is wont in such cases to set before him, wherein by his hellish malice he makes an infinite addition both to the already unnumbered multitude and to the too great heinousness of his sins, and would fain, if he will be led by his lying, cruelly misrepresent to his affrighted imagination every gnat as a camel, every mote as a molehill, every molehill as a mountain; every lustful thought as the most unclean act, every idle word as a desperate blasphemy, every angry look as an actual murder, every intemperate passion as an inexpiable provocation, every distraction in holy duties as an absolute rebellion, every transgression against light of conscience as a sin against the Holy Ghost. Nay, in this amazedness of spirit and disposition to despair, he is apt, even of his own accord, and with great eagerness, to arm every sin as it comes into his mind with a particular sting, that it may strike deep enough, and stick fast enough, in his already grieved soul. He employs and improves the excellency and utmost of his learning, understanding, wit, memory, to argue with all subtlety, with much sophistry, against the pardonableness of his sins and possibility of salvation. He wounds even his wounds with a conceit that they are incurable, and vexes his very vexations with refusing to be comforted. Not only crosses, afflictions, temptations, and all matter of discontentment; but even the most desirable things also in this life, and those which minister most outward comfort; wife, children, friends, gold, goods, great men's favours, preferments, honours, offices, even pleasures themselves, everything: whatsoever is within him, or without him, or about him; whatsoever he thinks upon, remembers, hears, sees, turn all to his torment. No marvel, then, though the terror of a wounded conscience be so intolerable." * Can any one doubt whether the foregoing is not a description of spurious remorse? Nor is such a kind of remorse confined to Christians. The Mahometan doctor, Malêc Ebn Ans, who is said to have "paid great regard to the traditions of Mohammed," and was remarkable for the conscientiousness of his instructions, was in his last illness found in tears, and upon being asked the reason of it, answered, "How should I not weep? and who has more reason to weep than I? Would to God that for every question decided by me according to my own opinion I had received so many stripes! Then would my accounts be easier. Would to God I had never given any decision of my own!" † It is also worthy of remark, that professed sceptics, even of the most intellectual order, have been equally liable to the inroads of remorse. Thus the ancient Epicureans themselves, who denied the intrinsic difference of human actions, all knowledge and concern of the gods about them, and consequently any future rewards and punishments, yet have left us the most graphic descriptions of remorse, as, for instance, Lucretius, lib. 3, v. 1024; and Mr. Hume describes remorse as one of the chief miseries common to all mankind.

VII. I have often observed spurious remorse to be a concomitant of incipient insanity, and of mental and bodily decay. In

* Bolton, sect. 1. Part xi.

† Sales, "Prelim. Dissert. to the Koran," sect. 8.

many cases of this kind the patients have complained that all the sins they ever committed, of thought, word, disposition, and action, even from their infancy, seemed revived to their recollections, and tormented their minds with incessantly repeated accusations. In not a few of these cases, however, the absolute impossibility of their having committed some of the sins which they said distressed their conscience, was obvious to their friends. Such sufferers have compared their sensations to a fire smouldering in their vitals, and have by their own involuntary movements pointed out the stomach, heart, liver, and hypochondrium as the origin of their agonizing perceptions. In high states of this affection, the patient believes that the supposed self-reproaches of his own heart are the effect of divine agency; as did Job when he complained to God, "Thou writest bitter things against me, and makest me to possess the iniquities of my youth;"* and as did the Roman emperor Tiberius, who, in his celebrated letter to the senate, attributed his remorse "to gods and goddesses eating him." In other. cases, the patient believes that Satan or that demons are the causes of his wretchedness, holds imaginary conversations with them, and, according, as it would seem, to the variations of the physical disorder, believes his infernal accusers to come or to go: for all morbid perceptions, or rather the perceptions suggested by morbid states of the body, have a tendency, in proportion to their intensity, to personify themselves to the consciousness; and the mental visions so created, may be even mistaken by the subjects of them for actual impressions on the several senses.

VIII. Spurious remorse often greatly exercises its tyranny over persons of fanatical, weak, and only partially cultivated mind, and of feeble will; or whose judgment is not the invariable rule of their ideas and conduct. Sir William Temple says, "An ingenious physician told me, that in the fanatic times, he found most of his patients so disturbed by troubles of conscience, that he was forced to play the divine with them before he could begin the physician."† Persons of this description, even during their comparative health, complain of "feeling as if they had done something dreadful," or, "as if something dreadful was going to happen to them;" are nearly always grieving and vexed about something or other, are easily offended, suspicious of being "slighted," and evince other indications of undue self-consciousness, or of inordinate attention to themselves. Their physical symptoms are frequently hysteria, palpitations of the heart, and hypochondriacal affections.

The last reason to be assigned for the merely morbid and physical origin of spurious remorse, is, that the subjects of it, though

* Job xiii. 26. † "On Health and Long Life." Works.

often conscious of its unreasonableness, rarely exert themselves in promoting their own relief. "I know," said one of the most amiable of these sufferers, the late poet James Montgomery, "that this is my own fault, and that I am an insane self-tormentor." * It is plain, however, that such a state of mind is not consistent with the *natural* effects and original intention of pain, which are to induce the patient to adopt, or to co-operate with, means for its mitigation. Neither do such states of mind yield to the intellectual consolations afforded by the Gospel, derived from its abundant representations of the infinite compassion of the Creator, and the perpetual and all-availing efficacy of the atonement as the medium of pardon for all confessed sin. Such sufferers, indeed, like the victims of morbid sentimentality, seem unwilling to part with their distresses. The writer, then, offers his conclusion from the foregoing reasons in the words of a well-known psychologist, that "religious enthusiasm and remorse, which often go hand in hand, are especially within the province of the physician." †

The first practical inference from the foregoing observations would seem to be, that owing to the possible morbid influences of bodily states upon the mind, the attempt never can be otherwise than precarious for any man to form a moral estimate either of his own general character, or of the character of any of his particular actions. It was possibly for such reasons that St. Paul considered "man's judgment of him a very small thing, and avoided judging himself."‡ It would seem equally difficult, for the same reasons, to form a correct judgment of a fellow-creature from the account given of him by himself. The majority of persons speak on all subjects beyond their immediate occupations, rather from their feelings than from a dispassioned judgment founded on facts. When, then, we are listening to a person's account of himself we are too probably listening only to an expression of his present feelings, and which are most likely more or less morbid, and certainly so in the case of every invalid. I am happy to find that the same inference has been formed by a writer of eminence, "On the Value of Feelings in Religion," in the following words—"And now from all that has been said, we can form no other conclusion than this,—that a man's feelings, or his state of mind, in *any* circumstances of his repentance and *future religious life*, possess no necessary and universal certainty. We might produce a number of passages from divines of high respect in confirmation of our opinions." §

* Memoirs of, by Holland and Everett, vol. ii.
† Feuchtersleben's "Medical Psychology." Sydenham Society, p. 136.
‡ 1 Cor. iv. 3.
§ "John Joachim Spalding," translated by Evans, 1827, pp. 248, 249.

2. It seems needful to be on our guard against what I must call the histrionic simulation by morbid feelings, of what are supposed to be, whether rightly or wrongly, those emotions, states of mind, &c., peculiarly desirable and proper. Whenever the mind, and especially of persons of excitable temperament, has formed to itself the *beau ideal* of any such state as admirable in itself or as worthy of imitation in others, the process of self-transformation into that state, by the minds of such persons, is neither tedious nor difficult. "Let me," says a writer already quoted, "discover unto you a mystery; but it is of iniquity and horrible hypocrisy. I have known some (would you think it?) who have counterfeited trouble of conscience; and made show without all truth or true touch of sundry temptations and spiritual distempers incidental only to the saints, and have for that purpose addressed themselves with much industry and noise, and had recourse many times to some spiritual physicians, with *many tears*, a heavy countenance, and other rueful circumstances expressing almost exactly the scruples, doubts, distrusts, complaints of such as are truly grieved in spirit and true of heart. O! the wonderful depth which lieth hid in the confluence of man's false heart! Such as these take upon them and lay aside terrors of conscience, as *players* do their apparel and parts."* I am emboldened by the foregoing statement to express my entire conviction that the "extraordinary," that is, emotional, piety of children and of very young persons, and especially their expressions of remorse, humility, &c., are of this *imitative* character: and further, that it is too possible for persons of any age, of a peculiar temperament, and under especial physical and social circumstances, to maintain an artificial character—in plain words—to act a part, even on the bed of death. I do not charge such children and dying persons with deliberate or systematic hypocrisy in the worse sense of the term, but I resolve the phenomena exhibited by them into the fascination of their own ideas, the flattery of their circumstances, and the influence of disease combining with the all but infinite delusiveness of morbid religious feelings. A popular instance of this kind, though of a melancholy aspect, may, I think, be found in the account of Francis Spira, who, according to the narrative of his remorse, lay on his bed *talking* and descanting on his condition to the bystanders in the following language—"I tell you there never was such a monster as I am: never was man alive a spectacle of such exceeding misery. I now feel God's heavy wrath, that burns like the torments of hell within me, and affects my soul with pangs unutterable. Verily desperation is hell itself. The gnawing worm of unquenchable fire, horror, *confusion*, and,

* Bolton, p. 222.

which is worst of all, desperation itself, continually tortures me. The truth is, never had mortal man such experience of God's anger and hatred against him as I have. The damned souls in hell endure not like misery, *therefore* I desire to *die.* Oh that some one would *let out this weary soul!* My state is worse than that of Cain and Judas." Yet the exciting cause of all these phrensies, was his recantation of his peculiar theological opinions from worldly considerations, for which opinions he had been previously conspicuous. Do not his references to his bodily sensations indicate that they were highly diseased? Is it compatible with the nature of real mental anguish, even of an ordinary kind, that the sufferer should describe it so eloquently? His sanity was indeed doubted by some intelligent observers at the time; but his ravings were considered by multitudes in that controversial age, as they are to this day—to have been a foretaste of the misery awaiting the apostate in a future state.

3. It must be inferred from the whole topic that much, to say the least, of what is now too commonly supposed to be pre-eminently a religious state of feeling, and the result of even divine agency, has a mere physical and even morbid origin; or to use the words of a fellow labourer in this department, that "the physical terrors of disease, the nervous anguish of a disordered brain *full of scaring images*, are absurdly held by many to be the actual sense and feeling of divine wrath, and the certain signs of complete repentance. But the *influence of the body on the mind in such cases*, and the *consequent violent emotions*, are too well known to be denied, except by those who know nothing of nature and its operations, and who choose to call everything supernatural which is unusual."* "It is also to be regretted, that many persons find it easier and even more agreeable to cultivate feelings of any kind, than to engage themselves in the close but noiseless study of the heart with its concealed impurities,—deliberate reflection on the great truths of religion and their reasonable grounds, due calculation of the gain or loss in our election between God and the present world, strict and incessant guard upon the conscience, the affections, and conduct,—*these* are too often smothered by the overwhelming sensations of enthusiasm; yet these alone are indispensable to true repentance, and violent emotions and excitements make not any necessary or essential part of it."† "The mind is, however, always disposed to strong emotions, and finds it more convenient to indulge than to explain them. It is loth to part with them, because it often has nothing with which to replace them. That spiritual instruction and guidance likewise is most welcome which gives least occupation to the thinking faculty,

* Spalding, p. 115. † Page 262, &c.

and withal is held to be the safest. Natural temperament, weakness and confusion of the mental powers, education, society, example, all contribute to the same effect."* I take courage from these confirmations of the preceding sentiments in this paper to avow my belief that what is called "popular preaching," too generally, owes its charms to its suitableness to the morbid susceptibilities of its admirers, and serves to alienate their hearts and minds still further from the practice of virtue, and from even the requisite attention to their temporal interests. The faithful and learned pastor will ever bear in mind the morbid condition of humanity, and avoid, above all things, awakening spurious remorse, agreeably to the memorable saying of ancient times, "sadness is the greatest enemy of God's servants;" and will accordingly shun the inculcation of superhuman attainments, artificial duties, giving romantic, loose, or inaccurate representations of repentance, and will patiently submit to the labour and self-denial which the observance of these precautions will involve, in regard of the most arduous part of his duties—the adaptation of the multifarious contents of the Scriptures to the real wants and substantial interests of his fellow men. It is important for all persons to beware of supposititious duties, because when once the sense of obligation is established in regard of any sort of feeling or line of conduct whatever, the moral faculty takes that sense of obligation under its keeping, and unfortunately, in the case of too many minds and temperaments, is too apt to embrace an erroneous rule more tenaciously, and to enforce it more rigorously, than a rule that is "holy, just, and good."

It now remains to offer some suggestions for the prevention and cure of spurious remorse. The obvious means of prevention is to make the development, and constant and inflexible exercise of the reason and judgment in regard of all subjects, the primary object of education, and to establish in the youthful mind a supreme regard to practical rectitude of both inward and outward action.

Children, too, should never be subjected to the influence of enthusiastic *preaching*. From the *services* and *offices* of the Church of England no danger can arise, for they all seem fitted to prevent the creation of spurious remorse and of every other morbid feeling.

It is also much to be wished that a knowledge of the evidences of natural and revealed religion were made a branch of education, and that the unrivalled works of Dr. Paley should be adopted as the text-book. Especially should the youthful mind be early imbued with a belief in the infinite goodness, mercy,

* Spalding, p. 254.

and reasonableness of the Creator in regard of all his dealings and intentions towards the human race, and of his requirements from them, and thoroughly enlightened respecting the provisions made by the atonement for the just and gracious exercise of these dispositions in the Deity towards the children of men; and carefully initiated into a firm and practical belief in the prevalence of confession of sins to God, as the immediate means of pardon and consolation.*

I earnestly dissuade parents from allowing indiscriminate religious reading to their children. Among the works to be placed in the parental *index prohibitory*, I find myself compelled by my convictions to name Doddridge's "Rise and Progress of Religion in the Soul," Bunyan's "Pilgrim's Progress," and all other works which describe and inculcate *a certain religious process* as necessary or even as desirable to be undergone, because children are often induced by the perusal of such works to force their feelings into a conformity with the representations they read; and because, "by such overstrained efforts, the mind is sure to lose its proper balance in some degree; and other contemplations are set aside, which would be of far more extensive and lasting benefit." †

The cure of the morbid physical and religious phenomena of Remorse, whenever apparent, depends primarily on the patient's restoration to bodily health and strength by those means indicated by his physical condition. During an appropriate course of medicine and diet, the acetate of morphia, unless forbidden by the especial circumstances of the case, is useful as an occasional sedative, until the healthy action of the viscera and brain can, by other means, be established. Mental remedies, or arguments addressed to the mind, will only be available in proportion as physical improvement advances. It seems advisable, as Dr. Feuchtersleben remarks with regard to any "fixed idea," for the attendants and friends "not to enter into it, by letting it pass unnoticed, and not appearing to think it worth while to refute it, or, when it can be done, pretending not to have heard or understood the patient;" or, in the case of morbid remorse, to change its direction by inspiring confidence in the infinite mercy of God.‡ Should pride, which *not unusually* attends this, as well as other forms of insanity, be suspected, it may be useful to lower the patient's undue self-importance by some such an expostulation as Elihu, misappropriately however, addressed to Job.§ Solitude and darkness should be avoided, and "occupation of the soberest kind, alternated with cheerful recreation, out of doors, and in a bracing atmosphere, must aid the direct religious

* 1 John i. 8, 9. † Spalding, p. 261. ‡ Pages 346, 347.
§ Job xxxv. 5–7.

instruction which may be practicable."* Since, too, spurious remorse is like some other diseases, periodical, its natural intervals and decline should be embraced as the most favourable opportunities for the employment of preventive means. The cure may be assisted by gradually drawing off the mind's attention from the feelings, and engaging it in the more active duties of life, and especially those duties which awaken a rational sense of self-interest. I subjoin below, more at length, the title of a work already quoted, of great value on the general subject.†

---

